# EMOTIONAL CO-REGULATION IN YOUNGER AND OLDER COUPLES’ DAILY LIVES: THE ROLE OF PSYCHOLOGICAL AVAILABILITY

**DOI:** 10.1093/geroni/igad104.1584

**Published:** 2023-12-21

**Authors:** Tabea Meier, Zilla Huber, Mike Martin, Andrea Horn

**Affiliations:** Northwestern University, Chicago, Illinois, United States; University of Zurich, Zurich, Zurich, Switzerland; University of Zurich, Zurich, Zurich, Switzerland; University of Zurich, Zurich, Zurich, Switzerland

## Abstract

Close relationships represent an important context for aging. Romantic partners can provide important resources for day-to-day individual and relational functioning by regulating their emotions together. Emotional co-regulation attempts are, however, not always successful and may depend on situational factors such as psychological availability, i.e., an individual’s current ability to be open and attentive to their partner’s needs. The present study examined emotional co-regulation in the daily lives of younger (N=62; 18-33 years) and older (N=56; 57-87 years) couples, focusing on the particular role of psychological availability. In a 21-day experience-sampling study, both participating partners reported their momentary emotional experiences, perceived psychological availability, relationship quality and (co)-regulation strategies three times a day. Results from dyadic multilevel modeling revealed that older and younger participants did not differ in the strategies they used to regulate their own and their partners emotions, but older couples showed greater emotional synchrony (i.e., coordinated changes between partners’ emotional experiences) throughout the study. When partners experienced greater psychological availability, they used more adaptive (e.g., positive humor, responsive touch) and fewer maladaptive (e.g., ruminative brooding) emotion regulation strategies. They also experienced more positive emotions and greater relationship quality when they were more psychologically available, and these associations were particularly evident among younger couples. The findings suggest that older partners were more attuned to each other’s emotions, but that their co-regulation depended less on psychological availability. This potentially speaks to more automatic co-regulation among older couples and opens the door for further investigations of situational factors that impact successful co-regulation.

